# RsaL-driven negative regulation promotes heterogeneity in *Pseudomonas aeruginosa* quorum sensing

**DOI:** 10.1128/mbio.02039-23

**Published:** 2023-10-16

**Authors:** Marta Mellini, Morgana Letizia, Lorenzo Caruso, Alessandra Guiducci, Carlo Meneghini, Stephan Heeb, Paul Williams, Miguel Cámara, Paolo Visca, Francesco Imperi, Livia Leoni, Giordano Rampioni

**Affiliations:** 1 Department of Science, University Roma Tre, Rome, Italy; 2 National Biofilms Innovation Centre, Biodiscovery Institute and School of Life Sciences, University of Nottingham, Nottingham, United Kingdom; 3 NBFC, National Biodiversity Future Center, Palermo, Italy; 4 IRCCS Fondazione Santa Lucia, Rome, Italy; Emory University School of Medicine, Atlanta, Georgia, USA

**Keywords:** *Pseudomonas aeruginosa*, quorum sensing, gene regulation, single cell analysis, heterogeneity, LasR, RsaL

## Abstract

In its canonical interpretation, quorum sensing (QS) allows single cells in a bacterial population to synchronize gene expression and hence perform specific tasks collectively once the *quorum* cell density is reached. However, growing evidence in different bacterial species indicates that considerable cell-to-cell variation in the QS activation state occurs during growth, often resulting in coexisting subpopulations of cells in which QS is active (quorate cells) or inactive (non-quorate cells). Heterogeneity has been observed in the *las* QS system of the opportunistic pathogen *Pseudomonas aeruginosa*. However, the molecular mechanisms underlying this phenomenon have not yet been defined. The *las* QS system consists of an incoherent feedforward loop in which the LasR transcriptional regulator activates the expression of the *lasI* synthase gene and *rsaL*, coding for the *lasI* transcriptional repressor RsaL. Here, single-cell-level gene expression analyses performed in *ad hoc* engineered biosensor strains and deletion mutants revealed that direct binding of RsaL to the *lasI* promoter region increases heterogeneous activation of the *las* QS system. Experiments performed with a dual-fluorescence reporter system showed that the LasR-dependent expression of *lasI* and *rsaL* does not correlate in single cells, indicating that RsaL acts as a brake that stochastically limits the transition of non-quorate cells to the quorate state in a subpopulation of cells expressing high levels of this negative regulator. Interestingly, the *rhl* QS system that is not controlled by an analogous RsaL protein showed higher homogeneity with respect to the *las* system.

**IMPORTANCE:**

Single-cell analyses can reveal that despite experiencing identical physico-chemical conditions, individual bacterial cells within a monoclonal population may exhibit variations in gene expression. Such phenotypic heterogeneity has been described for several aspects of bacterial physiology, including QS activation. This study demonstrates that the transition of non-quorate cells to the quorate state is a graded process that does not occur at a specific cell density and that subpopulations of non-quorate cells also persist at high cell density. Here, we provide a mechanistic explanation for this phenomenon, showing that a negative feedback regulatory loop integrated into the las system has a pivotal role in promoting cell-to-cell variation in the QS activation state and in limiting the transition of non-quorate cells to the quorate state in *P. aeruginosa*.

## INTRODUCTION

Many bacterial species utilize quorum sensing (QS) to coordinate social activities in response to changes in population density. QS is a cell-to-cell chemical communication process based on the extracellular release and perception of signal molecules. QS signal molecules are produced at basal levels at low cell density and accumulate in the medium as the bacterial population grows, until reaching a threshold concentration. The quorum is defined as the cell population density turning point at which signal molecules bind to and activate cognate transcriptional regulators or sensor kinases, leading to coordinated genetic reprogramming in all the cells of the population. A common feature of these systems is the positive effect exerted by the activated QS receptor on the production of the QS signal molecule, which generates a positive feedback loop that accelerates QS activation (1–3).

As one of the main systems involved in the control of bacterial collective behaviors, QS has long been regarded as a process homogeneously activated by the entire population in a highly synchronized way. However, gene expression analyses at the single-cell level suggest that this assumption is not the rule. Indeed, recent evidence indicates that QS activation is not always synchronous in single cells of a bacterial population and that the expression level of QS genes could considerably differ from one cell to another, sometimes resulting in a bifurcation of the population in quorate and non-quorate subpopulations (4–14). In some cases, heterogeneity has been shown to be transient and limited to the early phases of QS activation, with bacterial population subsequently converging to a homogeneous quorate state (11, 12). In other cases, inter-individual QS heterogeneity has been reported to persist also at high cell densities (4, 10, 15).

The investigation of cell-to-cell variability of QS activation is more complicated in bacteria possessing multiple and interconnected QS systems. As an example, *Vibrio harveyi* produces three different signal molecules, and the output QS response was found to be homogeneous only when all signals were produced at high levels, while heterogeneity arose in the absence of any one of the three signal molecules (16). A paradigmatic case of bacterial species possessing multiple QS systems is the opportunistic human pathogen *Pseudomonas aeruginosa*, in which three interconnected QS circuits, known as *las*, *rhl*, and *pqs*, are employed to finely modulate the expression of hundreds of genes in response to cell density and other environmental and metabolic cues. Notably, genes coding for several virulence factors and involved in biofilm formation is under the control of one or multiple QS systems in *P. aeruginosa*, highlighting QS synthase and receptor proteins as promising targets for the development of antivirulence drugs reducing *P. aeruginosa* pathogenicity (17, 18).

Heterogeneous QS activation has recently been addressed in *P. aeruginosa* (14, 19). Rattray and co-workers observed that activation of the QS-controlled gene *lasB* shows a heterogeneous and graded response to variations in the population density, indicating that there is no critical cell concentration triggering QS-dependent response (19). Moreover, their data indicate that the population diverges into subpopulations of quorate and non-quorate cells that also coexist at high cell density (19). Conversely, Jayakumar and colleagues found transient segregation of cells into discrete subgroups with distinct QS-related gene expression states at low cell density, with all the cells of the population converging to the quorate state at high cell density (14). Overall, despite no consensus being reached on the occurrence of non-quorate cells at high cell density, these seminal studies demonstrate that cell-to-cell variation in the QS activation state during *P. aeruginosa* growth significantly exceeds intrinsic gene expression noise (20, 21). At present, this remarkable finding lacks a solid molecular explanation.

**Fig 1:**
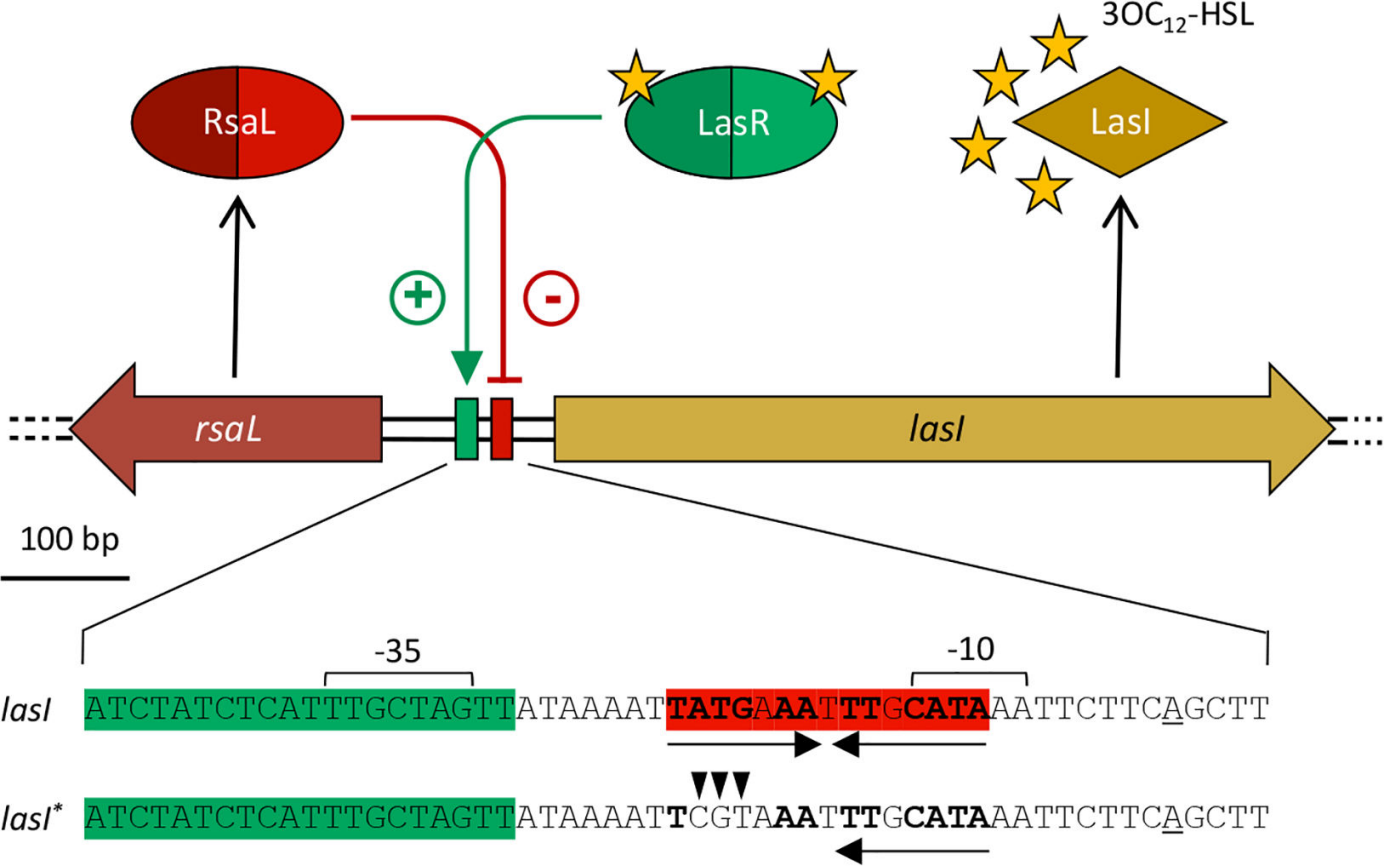
The *P. aeruginosa rsaL-lasI* gene *locus*. Schematic representation of the DNA region encompassing the *rsaL* and *lasI* genes in *P. aeruginosa* (37). Solid green arrow indicates activation (+); red T-line indicates negative regulation (−); the red and green boxes in the *rsaL-lasI* intergenic region indicate RsaL and LasR binding sites, respectively (32, 33). On the bottom, the *rsaL-lasI* bidirectional promoter region and the corresponding PlasI* variant (RsaL-binding negative) are depicted: the −35 and −10 regions are indicated; the LasR-binding site is green shadowed; the RsaL-binding site is red shadowed; transcriptional start site is underlined; repeats forming the palindromic sequence bound by RsaL are indicated by arrows; nucleotides whose substitution abrogates RsaL binding to the *rsaL-lasI* intergenic region are in bold; triangles indicate nucleotide substitutions introduced in the PlasI* variant.

Positive feedback loops, as those integrated into QS circuits, can generate or amplify gene expression heterogeneity (22–24), and also, negative feedback loops can favor cell-to-cell variation in gene expression (22, 25, 26). Youk and Lim showed *via* single-cell level analysis of a budding yeast strain engineered with a synthetic QS circuit that heterogeneity in the activation of this synthetic signaling system arose by integrating a positive feedback loop, which tends to amplify signal molecule production, with a negative regulatory system, which limits signal molecule availability (27). This regulatory architecture resembles the *P. aeruginosa las* QS system, in which the positive feedback loop generated by the LasR transcriptional regulator in complex with the *N*-(3-oxododecanoyl)-L-homoserine lactone (3OC_12_-HSL) signal molecule is counteracted by the negative feedback loop generated by RsaL (Fig. 1). Indeed, in the *las* system, the 3OC_12_-HSL signal molecule produced by the LasI synthase binds to and activates the intracellular receptor LasR, resulting in the regulation of diverse target genes (28–30). When interacting with the *rsaL-lasI* bidirectional promoter, the LasR/3OC_12_-HSL complex increases *lasI* transcription, thus generating the canonical positive feedback loop that enhances 3OC_12_-HSL production but also triggers transcription of the *rsaL* gene. RsaL, in turn, represses *lasI* transcription and its own expression when it binds to the *rsaL-lasI* bidirectional promoter on a palindromic sequence centered between the *lux*-box for LasR binding and the *lasI* transcriptional start site (Fig. 1) (31–34). Hence, on one hand, the LasR/3OC_12_-HSL complex promotes 3OC_12_-HSL synthesis, thus boosting its activation state and the QS response, while on the other, it limits 3OC_12_-HSL production and QS activation by stimulating the transcription of the QS negative regulator gene *rsaL*. Although apparently contradictory, such a regulatory mechanism, known as incoherent feed-forward loop, is widespread among biological systems (35). It is known to confer robustness to *lasI* gene expression with respect to fluctuations in the levels of LasR (36). Overall, this evidence prompted us to investigate the impact of RsaL on QS heterogeneity in *P. aeruginosa*.

By using single-cell level gene expression analyses, we show that the RsaL-driven negative regulation of *lasI* transcription increased cell-to-cell variation in the activation state of the *las* QS system and caused bifurcation of the population into subsets of quorate and non-quorate cells that also coexist at high cell density. Interestingly, the same phenotypes were not displayed by the *rhl* QS system that is not controlled by an incoherent feed-forward loop.

## RESULTS

### RsaL limits the transition of non-quorate cells to the quorate state

To investigate the impact of RsaL on the activation state of the *las* QS system at the single-cell level, a transcriptional fusion between the *lasI* promoter region, P*lasI*, and the *gfpmut3b* gene (herein referred to as *gfp*), named P*lasI::gfp*, was integrated into a neutral site in the chromosome of wild-type *P. aeruginosa* PAO1 and its isogenic Δ*rsaL* mutant. The P*lasI::gfp* fusion was also integrated in a PAO1 derivative with deletion of all the *las*, *rhl*, and *pqs* QS genes, named ΔQS (38), used as a control. P*lasI* was selected as this promoter is directly controlled by both the LasR/3OC_12_-HSL complex and RsaL (32, 33), and the LasR/3OC_12_-HSL-dependent increase of *lasI* transcription is generally considered as the triggering event for QS activation, with cells expressing *lasI* at higher levels relative to low cell density cells commonly defined as quorate cells (39). P*lasI* activity has been used as a proxy for *las* system activation also in other single-cell studies (14, 40, 41). Finally, the use of a chromosomally integrated reporter system avoids possible artifacts due to the unequal distribution and copy number of reporter plasmids among cells that could result in apparent gene expression heterogeneity and limits possible biases arising from the altered ratio between the intracellular levels of transcriptional regulators and the copy number of their target promoter.

**Fig 2:**
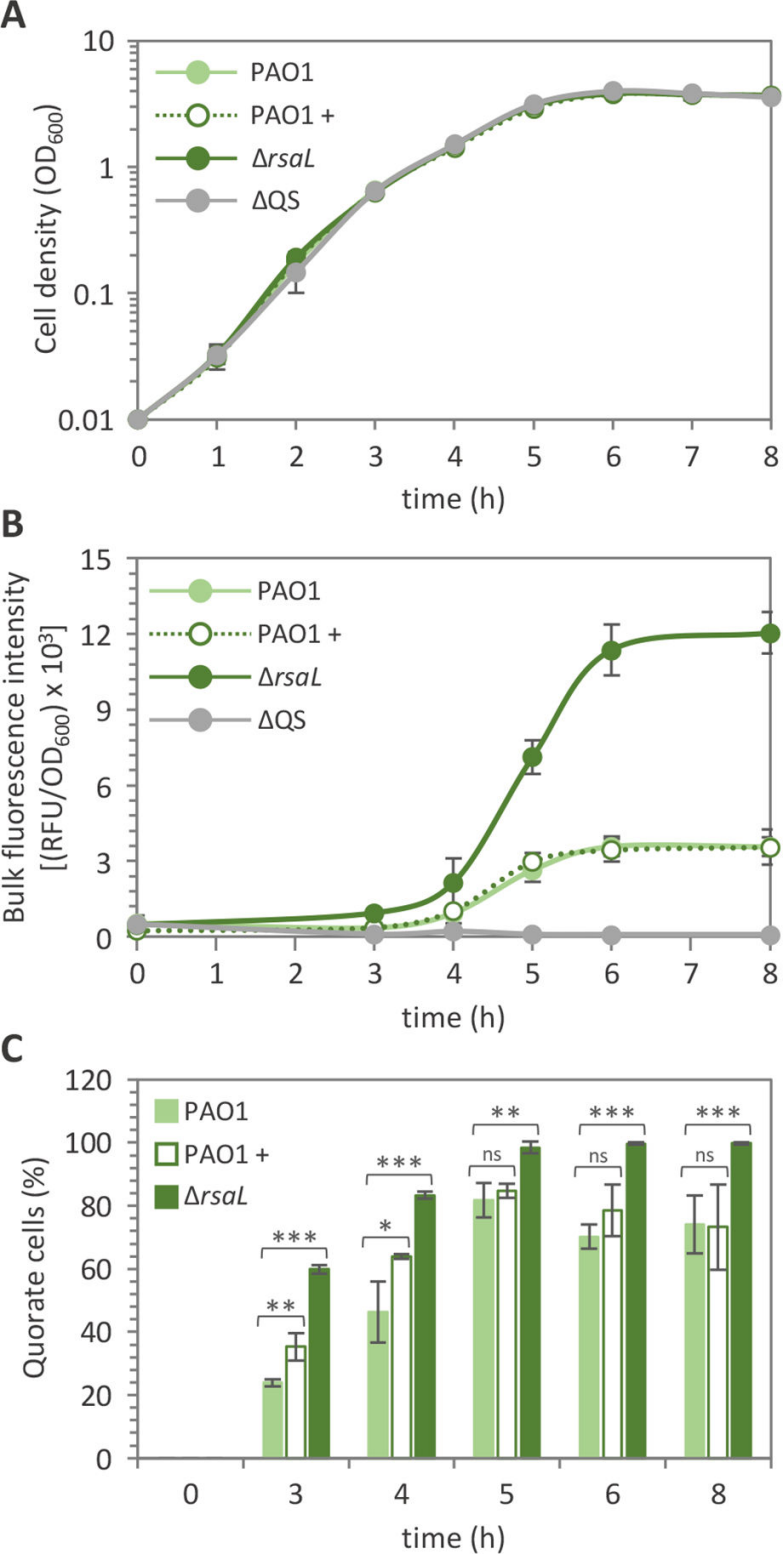
RsaL influences the proportion of quorate cells. (**A**) Growth curves, (**B**) bulk population analyses of P*lasI* activity, and (**C**) percentage of cells with active P*lasI* (quorate cells) in cultures of PAO1 incubated (+) or not with 10 µM 3OC_12_-HSL and of the indicated isogenic mutants, all carrying the P*lasI::gfp* fusion integrated into the chromosome. For panels **A** and **B**, means and standard deviations were obtained from three independent experiments. For panel **C**, the count of quorate cells for each sample at each time point was conducted on 2,250 cells from three biological replicates (750 cells each). *P < 0.05; **P < 0.01; ***P < 0.001; ns, not statistically significant.

At first, the reporter strains PAO1, Δ*rsaL*, and ΔQS carrying P*lasI::gfp* were validated by measuring P*lasI* activity in bulk populations. Growth curves of the tested strains were comparable in the rich medium lysogeny broth supplemented with 3-(*N*-morpholino)propanesulfonic acid (LB-MOPS) (Fig. 2A), confirming that QS is dispensable for growth under the conditions used in this study (MOPS was used to reduce 3OC_12_-HSL turnover caused by pH variation during growth). As expected, the Δ*rsaL* mutant showed higher P*lasI::gfp* activity compared to wild-type PAO1 during the whole growth curve, while promoter activity was negligible in the ΔQS mutant (Fig. 2B), in line with previous results obtained by using *lacZ* as the reporter gene (34, 36).

Then, P*lasI* activation has been analyzed in the reporter strains at the single-cell level by means of confocal microscope imaging. Images were analyzed with the ImageJ software for automatic counting of total cells in the brightfield and of quorate cells in the green florescent channel. Auto-fluorescence of ΔQS P*lasI::gfp* cells was considered as the baseline to clearly discriminate between quorate and non-quorate cells. In this context, it is important to highlight that *lasI* expression is expected to occur at a basal level at low cell density, thus contributing to the cell density-dependent accumulation of 3OC_12_-HSL in the medium. However, PAO1 cells expressing *lasI* at a level that is undistinguishable from the *lasI* expression level in ΔQS P*lasI::gfp* cells can be reasonably considered as non-quorate cells, despite they can still perceive 3OC_12_-HSL produced by neighboring cells. Conversely, the definition of quorate cells is somehow arbitrary, as a clear breakpoint in *lasI* expression level that allows unambiguous discrimination of quorate and non-quorate cells cannot be defined. To avoid underestimation of quorate cells, all the PAO1 P*lasI::gfp* cells showing higher fluorescence signals than ΔQS P*lasI::gfp* cells have been defined as quorate cells in this study. Based on this assumption, a small percentage (ca. 24%) of quorate cells was detectable in the PAO1 P*lasI::gfp* strain after 3-h growth (Fig. 2C), when the culture was in the exponential phase of growth (Fig. 2A). The percentage of quorate cells within the population gradually increased over time until it reached a fraction of ca. 80% at the onset of the stationary phase (5 h) and did not increase further (Fig. 2C). Addition of saturating levels of 3OC_12_-HSL (10 µM) to the growth medium did not significantly affect the percentage of quorate cells in the PAO1 P*lasI::gfp* culture, except for cells in the early exponential phase of growth (Fig. 2C). Notably, activation of the *las* system in the Δ*rsaL* P*lasI::gfp* strain occurred in about 60% of cells starting from the third hour of growth, and all the Δ*rsaL* cells expressed the P*lasI::gfp* fusion from the early stationary phase (i.e., from 5 h to 8 h) (Fig. 2C).

Comparable results were obtained in the PAO1, Δ*rsaL*, and ΔQS strains carrying a chromosomally integrated P*lasI::mCherry* translational fusion (Fig. S1), indicating that the results obtained by using the P*lasI::gfp* transcriptional fusion are unlikely to be artifacts due to intrinsic properties of the *gfp* reporter gene.

Taken together, these data corroborate previous evidence that single cells of *P. aeruginosa* population do not enter the quorate state in a synchronous way at a certain cell density (19). Indeed, an increasing fraction of cells gradually activated the *las* system as cell density increased, with a considerable fraction of cells remaining in the non-quorate state despite high cell density (OD_600_ ≈4). Bifurcation of the population relative to the QS activation state is unlikely due to incomplete saturation of LasR by 3OC_12_-HSL, given that exogenous provision of this signal molecule at saturating levels did not result in homogeneous QS activation. It should be noticed that, in the Δ*rsaL* strain, the quorate and non-quorate subpopulations coexisted only in the exponential phase of growth, while all cells converged to the quorate state in the stationary phase.

**Fig 3:**
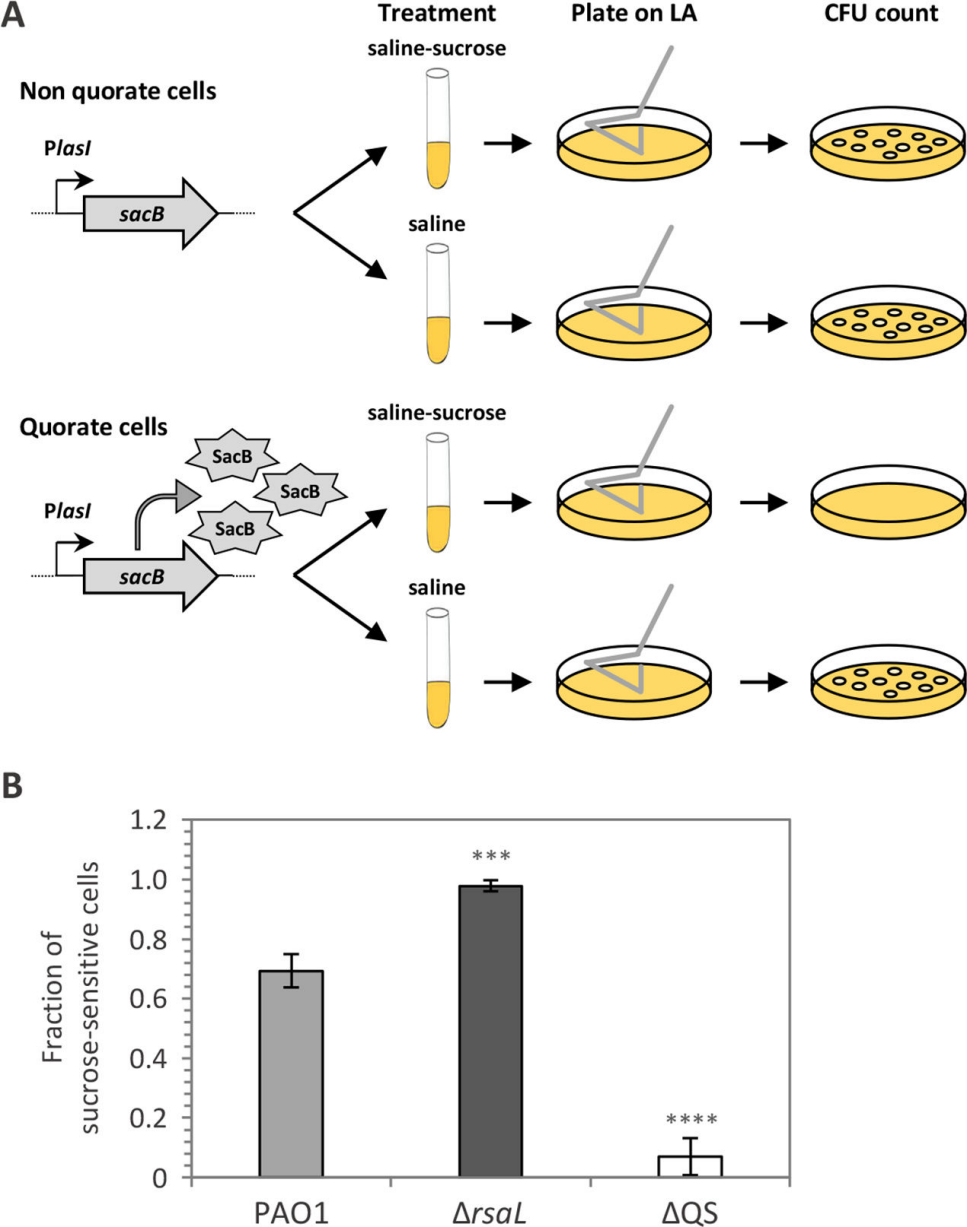
RsaL limits the transition of non-quorate cells to the quorate state at high cell density. (**A**) Schematic representation of the experiment performed on the PAO1, Δ*rsaL*, and ∆QS strains carrying the P*lasI::sacB* fusion integrated into the chromosome to investigate QS activation at high cell density. (**B**) Fraction of stationary-phase cells of PAO1, Δ*rsaL*, and ∆QS carrying the P*lasI::sacB* fusion that did not survive sucrose treatment. Means and standard deviations were obtained from three independent experiments. ***P < 0.001; ****P < 0.0001.

While the increase in the percentage of fluorescent cells carrying the P*lasI::gfp* or P*lasI::mCherry* fusion clearly demonstrates a graded transition of non-quorate cells to the quorate state as the cell density of the population increases, the possibility that confocal microscope detection limits could account for the apparent bifurcation of the bacterial population in quorate and non-quorate cells in stationary phase cannot be excluded. Hence, an alternative method was employed to investigate QS activation at high cell density. A construct for P*lasI*-dependent control of *sacB* expression, named P*lasI::sacB*, was integrated into the chromosome of the PAO1, Δ*rsaL*, and ΔQS strains. The *sacB* gene encodes for the levansucrase enzyme, which converts sucrose to levan, a toxic substance that accumulates into the periplasm and causes cell lysis (42, 43). Based on the idea that quorate cells triggering *sacB* expression should undergo lysis in the presence of sucrose, while non-quorate cells that express *sacB* at basal levels should be insensitive to sucrose exposure, we compared the ability to survive sucrose treatment of the PAO1, Δ*rsaL*, and ΔQS strains carrying the P*lasI::sacB* fusion (Fig. 3A). While the ability to discriminate fluorescence positive and negative cells can be influenced by the characteristics of the microscope and the experimental settings employed for the analysis, the *sacB*-based assay employed in this study allows unbiased investigation of QS heterogeneity by linking the quorate or non-quorate state to a binary phenotype, i.e., death or survival in the presence of sucrose, respectively.

Briefly, reporter strains grown to stationary phase (6 h of growth) were incubated for 1 h in sterile saline supplemented or not with 10% (wt/vol) sucrose before CFU counting on LB agar plates. This analysis revealed that ca. 30% of PAO1 cells in the stationary phase did not activate the P*lasI::sacB* fusion at levels sufficient to cause cell death, while none of the ∆*rsaL* cells carrying the P*lasI::sacB* fusion survived sucrose treatment in the same conditions (Fig. 3B). As expected, the CFU count of sucrose-treated and sucrose-untreated ΔQS P*lasI::sacB* cells was comparable (Fig. 3B), as P*lasI* is active at a basal level in this mutant strain (Fig. 2B).

These results confirm that discrete subpopulations of quorate and non-quorate cells coexist even in conditions of high cell density and that RsaL has a primary role in limiting the transition of non-quorate cells to the quorate state in the *P. aeruginosa* population.

### RsaL increases cell-to-cell variation in *las* QS expression

It has been previously reported that quorate cells exhibit various degrees of *lasI* expression during growth (14). To assess population heterogeneity, we made use of single-cell analyses to determine the impact of RsaL on P*lasI* response. Confocal microscopy images were used to quantify fluorescence emission from each cell as a proxy for P*lasI* activation. These data enabled the calculation of the coefficient of variation (CV), commonly used as an index of heterogeneity (14, 16). Hence, CV was calculated by dividing the standard deviation obtained from all fluorescence intensity values of single cells by the mean of the same fluorescence intensity values. Therefore, high CV values reflect high levels of heterogeneity and vice versa.

**Fig 4:**
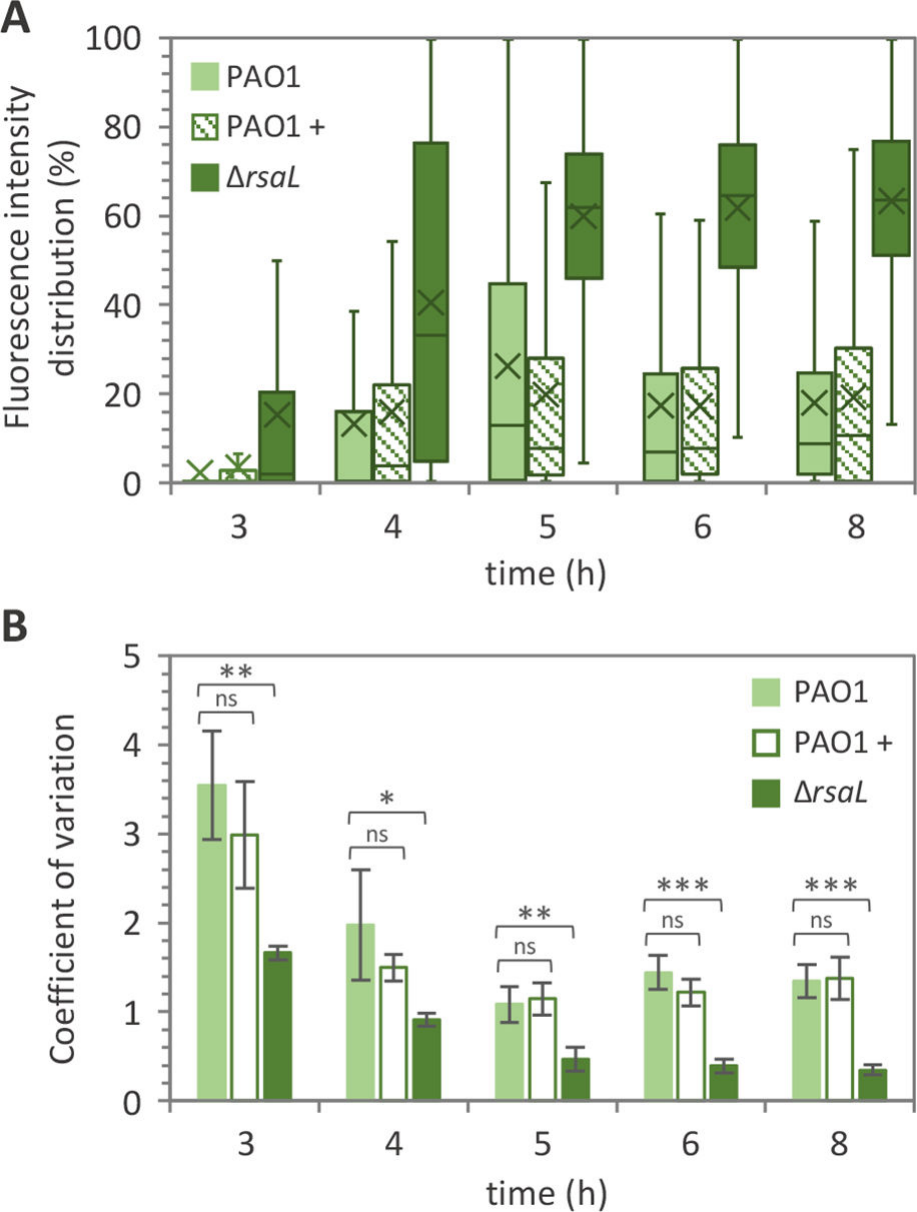
RsaL increases heterogeneity of QS activation. (**A**) Box plot graph showing fluorescence distribution and (**B**) coefficient of variation from single cells of PAO1 incubated (+) or not with 10 µM 3OC_12_-HSL and its isogenic Δ*rsaL* mutant, both carrying P*lasI::gfp* fusion integrated into the chromosome. For panel **A**, fluorescence intensity is given as percentages relative to the Δ*rsaL* P*lasI::gfp* cell showing the highest fluorescence intensity after 8 h of growth, considered as 100%. Mean P*lasI* activity derived from fluorescence intensity values measured in single cells is indicated with an X in the box plots. The horizontal lines in the box plots represent the median values. For each biological replicate and at each time point, fluorescence quantification has been conducted on 750 cells per strain/condition. A representative data set from one out of three biological replicates is shown. For panel **B**, means and standard deviations were obtained from three biological replicates (750 cells each). *P < 0.05; **P < 0.01; ***P < 0.001; ns, not statistically significant.

This analysis demonstrated that quorate cells display a wide distribution of P*lasI* activities that were non-significantly affected by the exogenous provision of 3OC_12_-HSL and that fluorescence intensity values are higher, on average, in the ∆*rsaL* mutant relative to PAO1 (Fig. 4A and Fig. S2). Interestingly, the trend of mean P*lasI* activity during growth (derived from fluorescence intensity values measured in single cells and indicated with an “X” in the box plots of Fig. 4A) was comparable to the trend of P*lasI* activity measured in bulk populations (Fig. 2B). For all the tested strains, CV values showed a tendency to decrease proceeding with growth (Fig. 4B), confirming that QS activation moves to higher synchronicity at higher cell densities. Supplementation with 3OC_12_-HSL did not significantly alter P*lasI* heterogeneity levels in PAO1, while the Δ*rsaL* population showed lower CV values compared to PAO1 at all time points (Fig. 4B). Similar results were obtained by measuring mCherry fluorescence in single cells of the PAO1 and Δ*rsaL* strains carrying the P*lasI::mCherry* fusion (Fig. S3).

These findings indicate that, in addition to its ability to induce bifurcation of the population in quorate and non-quorate cells, RsaL also increased heterogeneity in P*lasI* activation levels.

### The effect of RsaL on QS heterogeneity relies on its ability to bind to the *lasI* promoter region

RsaL is a global transcriptional regulator that directly affects the expression of several genes in addition to *lasI* (34, 44). Hence, RsaL could impact on the transition of non-quorate cells to the quorate state both directly, by binding to its target sequence on the *lasI* promoter region, and indirectly, by modulating the expression of still unknown molecular actors involved in QS regulation. To investigate this issue, a new set of biosensor strains in which *gfp* expression is under the control of a mutated P*lasI* variant impaired in RsaL binding, herein referred to as PlasI*, was generated. The palindromic sequence required for RsaL binding to the *lasI* promoter region was disrupted in PlasI* by substitution of three nucleotides whose mutation was previously shown to abrogate RsaL binding to DNA (Fig. 1) (45).

**Fig 5:**
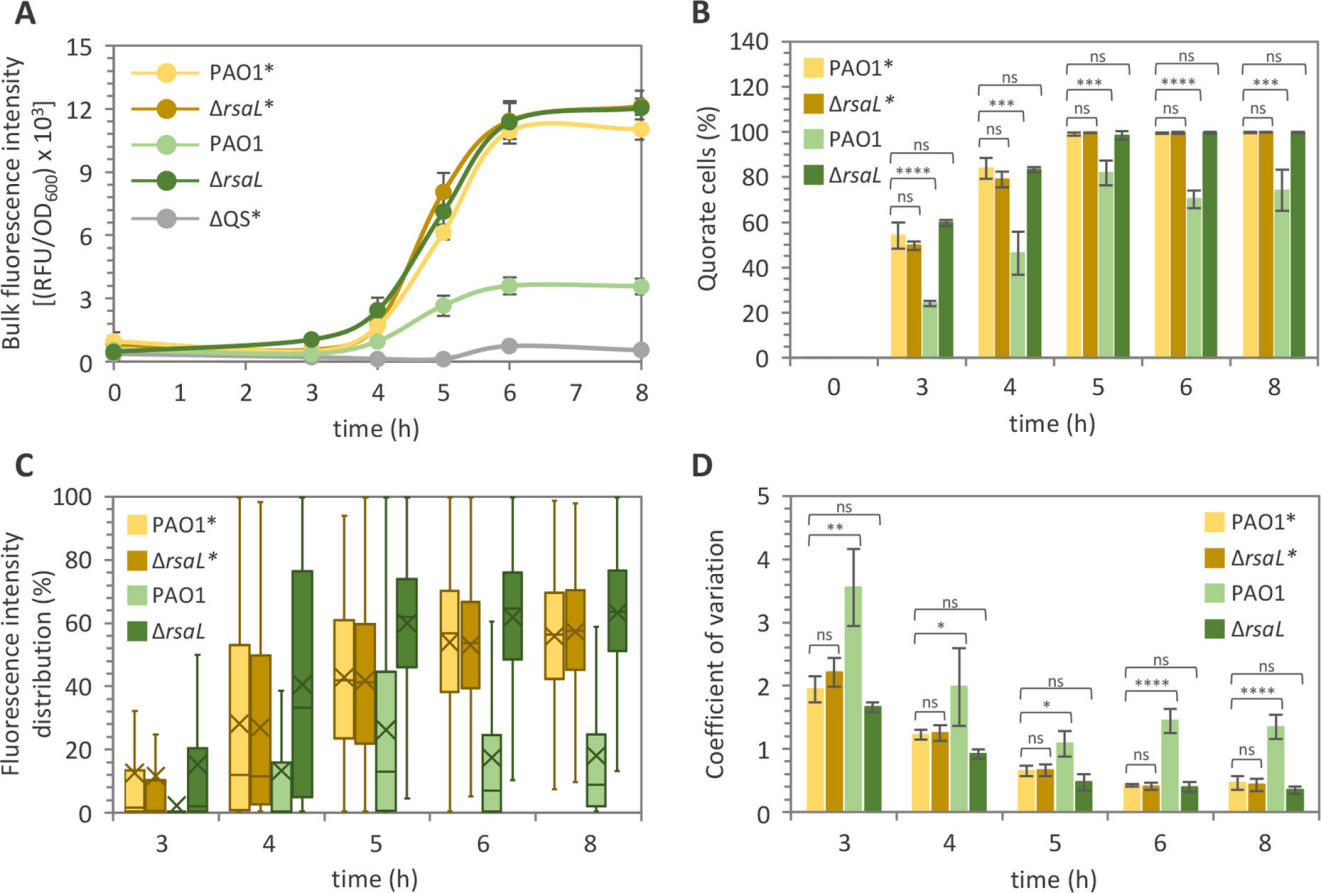
Heterogeneity of QS activation requires direct binding of RsaL to the *lasI* promoter region. (**A**) Bulk population analyses of P*lasI* and PlasI* activity and (**B**) fraction of cells activating P*lasI* or PlasI* in cultures of the PAO1 and ∆*rsaL* strains carrying the P*lasI::gfp* or PlasI*::gfp fusion integrated into the chromosome. (**C**) Representative box plot graph showing fluorescence distribution and (**D**) coefficient of variation from single cells of the PAO1 and ∆*rsaL* strains carrying the P*lasI::gfp* or PlasI*::gfp fusion integrated into the chromosome. For panel **A**, the average of three independent experiments is reported with standard deviations. For panels **B** and **D**, means and standard deviations were obtained from three biological replicates (750 cells each). For panel **C**, fluorescence intensity is given as percentages relative to the Δ*rsaL* P*lasI::gfp* cell showing the highest fluorescence intensity after 8 h of growth, considered as 100%. Mean P*lasI* and PlasI* activity derived from fluorescence intensity values measured in single cells is indicated with an X in the box plots. The horizontal lines in the box plots represent the median values. For each biological replicate and at each time point, fluorescence quantification has been conducted on 750 cells per strain. A representative data set from one out of three biological replicates is shown. *P < 0.05; **P < 0.01; ***P < 0.001; ****P < 0.0001; ns, not statistically significant.

Interestingly, bulk fluorescence emission (Fig. 5A), percentage of quorate cells (Fig. 5B), distribution of fluorescence intensity in single cells (Fig. 5C), and gene expression heterogeneity (CV values; Fig. 5D) were comparable between the PAO1 and ∆*rsaL* strains carrying the PlasI*::*gfp* fusion along the whole growth curve, resembling the behavior of the P*lasI::gfp* fusion in the ∆*rsaL* mutant.

Taken together, these data indicate that RsaL affects cell-to-cell variation in QS activation level and the transition of non-quorate cells to the quorate state by directly interacting with the *lasI* promoter region, excluding any role for other RsaL-controlled genes.

### Expression of *rsaL* is high in non-quorate cells and low in quorate cells

The adjacent *lasI* and *rsaL* genes are divergently oriented in the *P. aeruginosa* chromosome (37). Expression of the *lasI* and *rsaL* genes from the *rsaL-lasI* bidirectional promoter is apparently characterized by a high degree of regulatory symmetry. Indeed, the LasR/3OC_12_-HSL complex triggers transcription of both *lasI* and *rsaL* when interacting with its *lux-*box target sequence, while binding of RsaL to a unique site adjacent to the *lux-*box abrogates both *lasI* and *rsaL* expression (Fig. 1) (32, 34).

**Fig 6:**
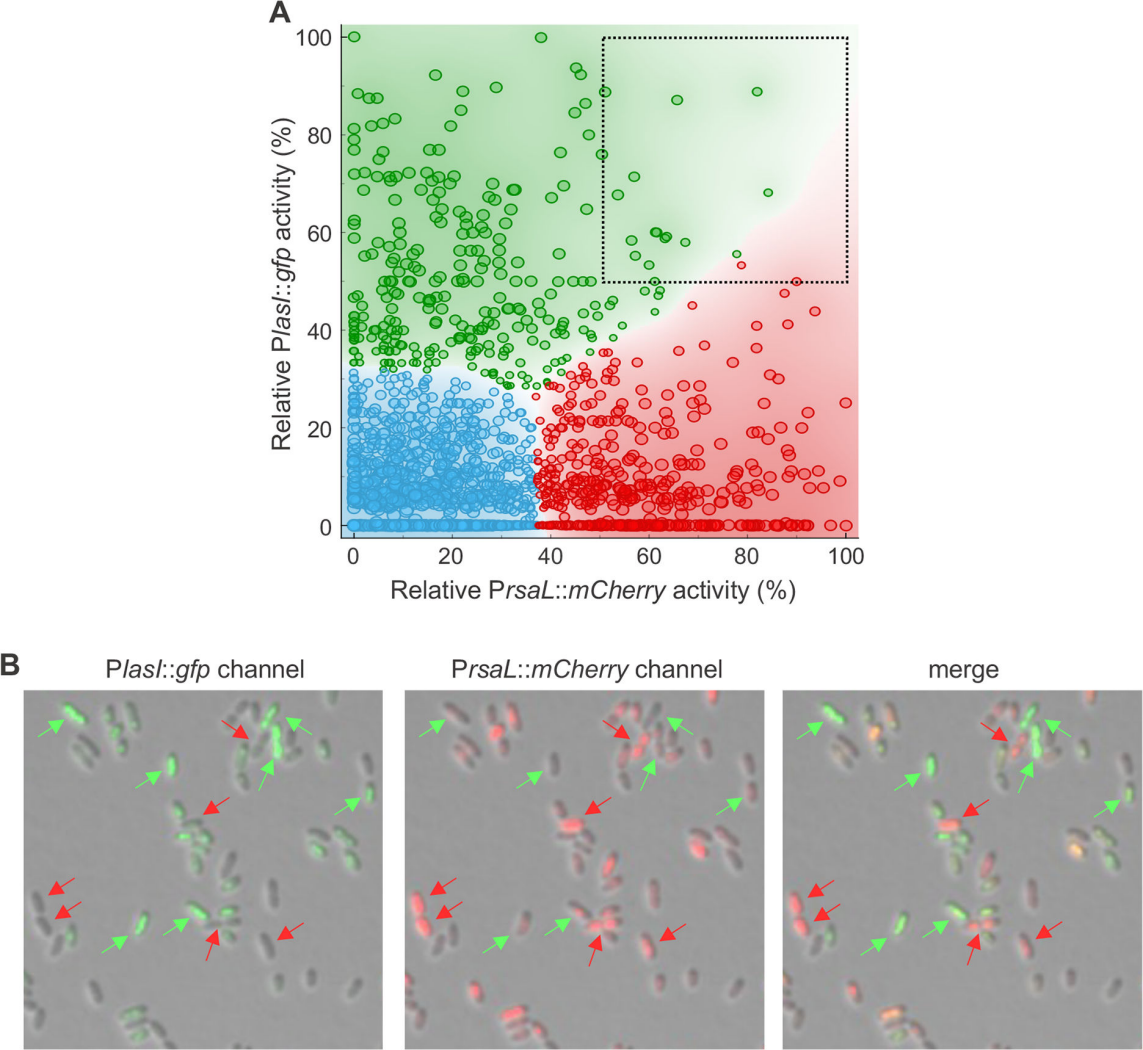
The expression levels of *lasI* and *rsaL* do not correlate in single cells. (**A**) Fluorescence intensities of GFP and mCherry measured in 1,500 cells from the PAO1 P*lasI::gfp*-P*rsaL::mCherry* population grown to stationary phase. The fluorescence intensities are reported as percentages of the maximum measured intensity for GFP and mCherry channels, respectively. The data were grouped (blue, red, and green) using k-means clustering algorithm; the size of each point in the plot is proportional to its silhouette score in each cluster. Weaker scores were observed on the boundary between clusters. The cells with relative fluorescence intensity of both GFP and mCherry greater than 50% are in the top-right dashed square of the plot. (**B**) Representative images of stationary-phase PAO1 cells carrying the P*lasI::gfp*-P*rsaL::mCherry* dual reporter construct. Left image, merge of GFP and brightfield channels; center image, merge of mCherry and brightfield channels; right image, merge of brightfield, GFP, and mCherry channels. Green arrows indicate cells expressing high levels of GFP and low levels of mCherry; red arrows indicate cells expressing high levels of mCherry and low levels of GFP.

In this regulatory framework, an attempt has been made to identify an asymmetric element generating heterogeneity in QS activation. To this purpose, the dual reporter mP*lasI::gfp*-P*rsaL::mCherry* construct was integrated into the chromosome of the wild-type PAO1 and its isogenic ΔQS mutant. The mP*lasI::gfp*-P*rsaL::mCherry* construct allows simultaneous monitoring of both P*lasI* and P*rsaL* activities in single cells by measuring their fluorescence intensity in the green and red channels, respectively. Also in this case, the fluorescence emission from single cells of the ΔQS P*lasI::gfp*-P*rsaL::mCherry* strain was considered as the baseline. The GFP vs mCherry fluorescence intensities of PAO1 cells, normalized to the respective maximum fluorescence intensities, are reported in Fig. 6A. The data broadly distributed in the plot are grouped (k-means clustering algorithm) in three clusters of cells which maximize the silhouette score: (i) a cluster close to the origin with low/intermediate GFP and mCherry fluorescence intensities (blue cluster); (ii) a cluster with high-GFP/low-mCherry fluorescence intensities (green cluster); (iii) a cluster with high-mCherry/low-GFP fluorescence intensities (red cluster). Visually, no correlation is observed between GFP and mCherry fluorescence signals. In the case of a positive correlation, the points should be arranged with an increasing trend in the graph, while in the case of a negative correlation, one would expect them to be arranged with a decreasing trend. Indeed, the correlation between GFP and mCherry fluorescence intensities was very close to zero (Pearson coefficient: ρ = −0.09). Accordingly, cells in which P*lasI* and P*rsaL* are both highly activated were rare, as only ca. 1% of the cells in the population showed relative fluorescence signals >50% for both GFP and mCherry (top-right square in Fig. 6A). This clustering was evident also when cells were visually inspected, as PAO1 P*lasI::gfp*-P*rsaL::mCherry* cells with high levels of GFP (i.e., quorate cells) showed tendency to have low or no detectable mCherry fluorescence, and vice versa (Fig. 6B). This analysis demonstrates that the LasR/3OC_12_-HSL complex does not simultaneously and equally contribute to both *lasI* and *rsaL* transcription in all the cells of the population. Rather, stochastic regulation occurs causing the alternative prevalence of either *lasI* or *rsaL* expression in distinct subpopulations of cells, promoting cell-cell heterogeneity that is reinforced by the negative regulation exerted by RsaL on *lasI* transcription, ultimately leading to the segregation of cells into discrete subgroups of quorate and non-quorate cells.

### Activation of the *rhl* system shows low heterogeneity

An intriguing question is whether heterogeneity occurs also in the activation state of QS systems that are not controlled by a negative feedback loop. In this context, the *rhl* QS circuit of *P. aeruginosa* is an appropriate test-bed system, as similarly to the *las* QS circuit, its activation relies on the positive feedback loop generated by the RhlR/*N*-butanoyl-l-homoserine lactone (C_4_-HSL) complex (46), but it is not repressed by RsaL or any analogous regulator (34, 44, 47) (Fig. S4).

**Fig 7:**
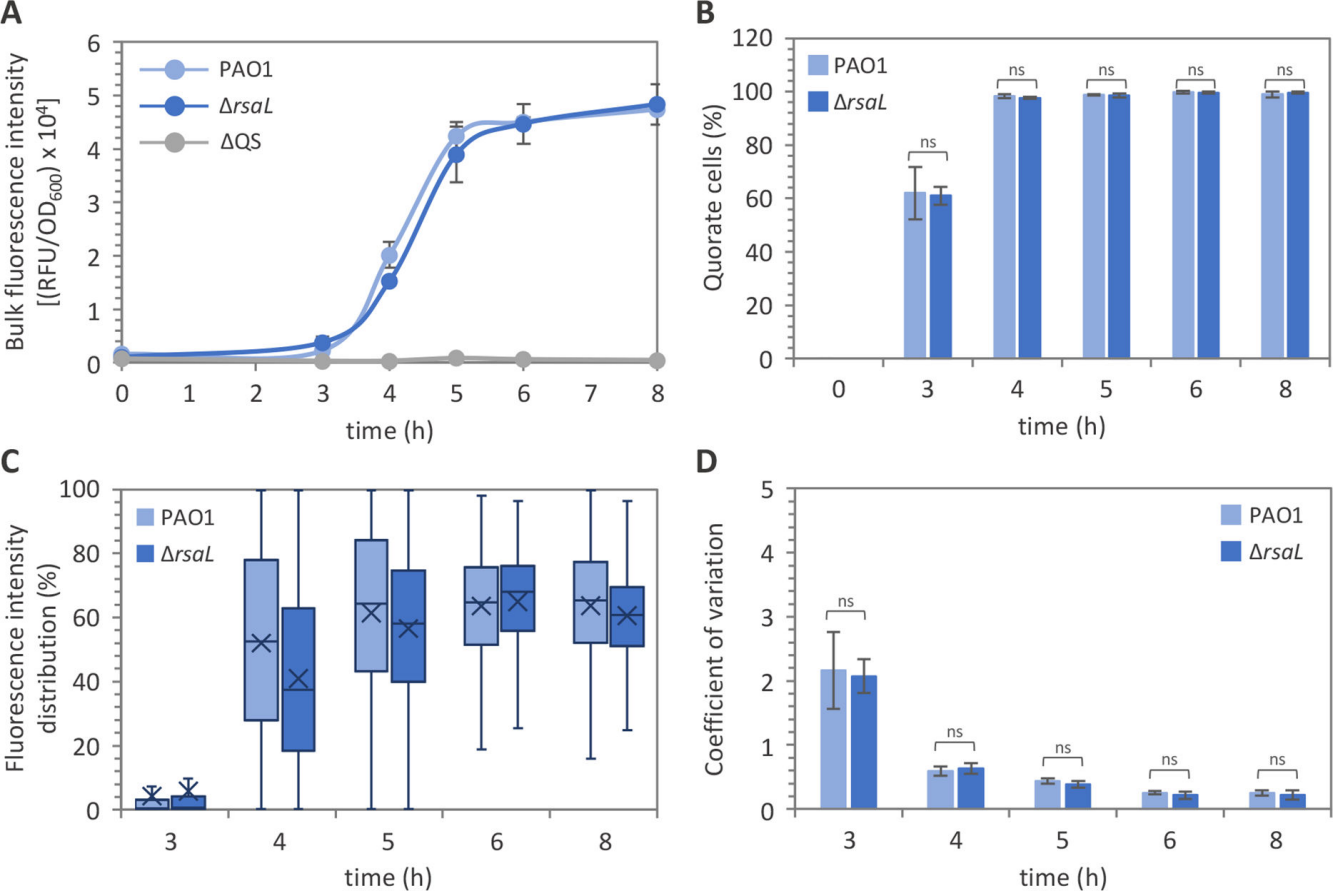
The *rhl* system exhibits higher homogeneity than the *las* system. (**A**) Bulk population analyses of P*rhlI* activity and (**B**) fraction of cells activating P*rhlI* in cultures of the indicated strains carrying the P*rhlI::gfp* fusion integrated into the chromosome. (**C**) Box plot graph showing fluorescence distribution and (**D**) coefficient of variation from single cells of the PAO1 and ∆*rsaL* strains carrying the P*rhlI::gfp* fusion integrated into the chromosome. For panel **A**, the average of three independent experiments is reported with standard deviations. For panels **B** and **D**, means and standard deviations were obtained from three biological replicates (750 cells each). For panel **C**, fluorescence intensity is given as percentages relative to the PAO1 P*rhlI::gfp* cell showing the highest fluorescence intensity after 8 h of growth, considered as 100%. Mean P*rhlI* activity derived from fluorescence intensity values measured in single cells is indicated with an X in the box plots. The horizontal lines in the box plots represent the median values. For each biological replicate and at each time point, fluorescence quantification has been conducted on 750 cells per strain. A representative data set from one out of three biological replicates is shown. ns, not statistically significant.

Here, we made use of an integrative plasmid carrying the P*rhlI::gfp* transcriptional fusion to monitor the activation state of the *rhl* QS system during the growth of individual PAO1 and ∆*rsaL* cells. Bulk fluorescence analysis confirmed the predicted response of the construct, as the P*rhlI::gfp* fusion was induced during growth in the PAO1 strain, but not in the ∆QS mutant (Fig. 7A). Interestingly, all cells of the PAO1 population activated the P*rhlI* promoter already after 4 h of growth (Fig. 7B). Moreover, even if P*rhlI* activity showed a certain degree of heterogeneity among cells (Fig. 7C; Fig. S5), CV values derived from fluorescence quantification in single cells were lower in PAO1 P*rhlI::gfp* relative to PAO1 P*lasI::gfp* (compare Fig. 4B and Fig. 7D). In accordance with previous studies showing that RsaL does not affect *rhlI* transcription (34, 47), none of the tested P*rhlI::gfp* parameters was altered in the ∆*rsaL* mutant relative to wild-type PAO1 (Fig. 7; Fig. S5).

Overall, these data indicate that the activation of the *rhl* system exhibits higher homogeneity and synchronicity at the single-cell level compared to the *las* system. Differences in the activation dynamics of the *las* and *rhl* QS systems are likely due to the lack of a built-in negative regulator in the *rhl* system that can fulfil RsaL-like functions.

## DISCUSSION

Over the years, single-cell level studies revealed that heterogeneity in the activation of QS systems is not an unusual feature (48–50). However, the molecular basis of such heterogeneity has seldom been investigated. Here, we identify RsaL as a key molecular actor determining cell-to-cell variation of *lasI* expression in *P. aeruginosa* and demonstrate that binding of this negative regulator to the *rsaL-lasI* bidirectional promoter is pivotal for heterogeneous activation of the *las* QS system.

In line with findings of Rattray and collaborators, which monitored the QS-regulated P*lasB* promoter at the single-cell level under conditions of varying carbon availability (19), we found that *las* system activation was graded with respect to increasing levels of cell densities. Such recent evidence about graded QS induction in *P. aeruginosa*, obtained via single-cell level analysis, should lead to reconsidering the canonical vision of the threshold-dependent QS activation, as in some cases, QS might not necessarily be characterized by an “OFF/ON” status determined by a specific level of cell density.

We also found that the PAO1 population tends to be less heterogeneous with respect to P*lasI* activation proceeding with growth, in accordance with reference 14. However, a certain level of heterogeneity also persisted into the stationary phase, where a significant fraction of cells did not activate the P*lasI* promoter. The elegant analyses performed by Rattray and collaborators in minimal M9 medium supplemented with MOPS showed a similar pattern of activation of the *lasB* promoter for the higher cell densities they considered (19), in line with our findings showing the coexistence of quorate and non-quorate cells in stationary phase populations. Since the maximum OD_600_ reached by *P. aeruginosa* populations used by Rattray and colleagues was <0.8 (19), it may not have been sufficient to fully trigger QS. Here, we conducted our analyses in the rich medium LB-MOPS, in which PAO1 cultures reached an OD_600_ of ca. 4 in the stationary phase, and at such high cell densities, we still observed the coexistence of quorate and non-quorate cells. The fact that not all the cells of a population enter the quorate state at high cell densities is not limited to *P. aeruginosa*. Indeed, this phenomenon has also been described for *Pseudomonas syringae* pv. *syringae*, *Xanthomonas campestris*, *V. harveyi*, and *Sinorhizobium fredii* (4, 9, 10).

In this study, neither the quorate cell fraction nor the degree of heterogeneity was particularly affected when saturating levels of synthetic 3OC_12_-HSL were added to *P. aeruginosa* cultures. Also, *P. syringae* and *X. campestris* have been demonstrated to retain QS heterogeneity following exogenous signal molecule addition (10). On the other hand, in *S. fredii* (9) and *V. harveyi* (4), QS synchronization occurred in response to external signal molecule provision, leading the authors to hypothesize that, in these bacteria, QS heterogeneity could be ascribed to an unsaturated state of signal molecule receptor. In *P. aeruginosa*, saturating 3OC_12_-HSL levels do not shift the population to a homogeneously quorate state, meaning that unsaturated LasR levels cannot account for heterogeneity in QS activation. Moreover, we previously showed that the incoherent feed-forward loop integrated into the *las* system confers robustness with respect to fluctuations in LasR levels to promoters controlled by both LasR/3OC_12_-HSL and RsaL (36). Hence, it is reasonable to hypothesize that the incoherent feed-forward loop generated by LasR and RsaL could confer robustness to P*lasI* activity also with respect to fluctuations in 3OC_12_-HSL levels. Furthermore, the inability of exogenous signal molecule provided at saturating levels to induce QS activation at the onset of bacterial growth could rely on the action of the QscR, QslA, and/or QteE anti-activators, which keep the LasR regulator in an inactive state not competent for 3OC_12_-HSL binding at low cell density (51–54). The transcriptional regulator MvaT could also play a role in this phenomenon, as exogenous 3OC_12_-HSL can significantly advance the expression of *las*-controlled genes in a *mvaT* mutant but not in wild-type PAO1 (55).

Our work describes for the first time the involvement of RsaL in controlling the transition of non-quorate cells to the quorate state. Likewise, the LuxO repressor, through destabilization of the *luxR* transcript, was shown to increase heterogeneity in bioluminescence emission in *V. harveyi* (4). Transient co-existence of quorate and non-quorate cells in the ∆*rsaL* mutant during the exponential phase of growth could possibly be attributed to intrinsic transcriptional noise. However, the possibility that factors other than RsaL could contribute to cell-to-cell variation in the activation state of the *las* QS system at low cell density should be considered. Interestingly, evidence for heterogeneous *lasR* gene expression has been provided during exponential growth (14), indicating that the positive feedback loop generated by the LasR/3OC_12_-HSL complex may contribute to *las* system heterogeneity at the onset of QS activation. In this context, it is worth mentioning that a positive feedback loop has been shown to increase heterogeneity of QS activation in *Bacillus subtilis* (24). However, a study from Scholz and Greenberg reported that the *las* QS response shows greater synchrony in wild-type *P. aeruginosa* than in an engineered strain lacking the positive feedback loop controlling 3OC_12_-HSL synthesis, in which the signal molecule was constantly produced regardless of the population cell density (40).

It is relevant to note that population-based and single-cell level analyses could lead to different interpretations of the same phenomenon. According to the previously proposed model of *las* system activation and QS homeostasis (34), the LasR/3OC_12_-HSL complex can drive transcription of the *rsaL* and *lasI* genes at the quorate cell density. The LasR/3OC_12_-HSL-dependent positive feedback loop increases both 3OC_12_-HSL synthesis and RsaL levels, to the point that RsaL interacts with its DNA-binding site on the *rsaL-lasI* bidirectional promoters. This abrogates transcription of both *lasI* and *rsaL*, thus limiting 3OC_12_-HSL production to productive levels at high cell density. Although this was the conclusion that clearly emerged when *las* QS activation was analyzed at the population level, the single-cell level analyses performed here are consistent with an alternative mechanistic model. When the LasR/3OC_12_-HSL complex binds to the *rsaL-lasI* bidirectional promoter, stochastic events could result in asymmetric transcription of either *lasI* or *rsaL* in individual cells instead of homogeneous expression of these genes in the whole population. The negative regulation exerted by RsaL on *lasI* transcription could reinforce the variance between RsaL and LasI production among cells, further limiting QS activation in the proportion of cells characterized by high *rsaL* expression levels. In this context, the uneven LasR/3OC_12_-HSL-dependent transcription of *lasI* and *rsaL* appears to be a primary driver of QS heterogeneity. The absence of correlation between *lasI* and *rsaL* expression in single *P. aeruginosa* cells supports this model.

Activation of the *rhl* system at the single-cell level follows a different trend compared to the *las* system, as this QS system appeared to be homogeneously activated by all cells starting from the exponential growth phase and was unaffected by RsaL. This finding was unanticipated, as it is generally accepted that the *las* system exerts positive transcriptional control over both *rhlR* and *rhlI* (46). However, it must be considered that previous studies showed deregulation of the *las* QS system but not of the *rhlR* and *rhlI* transcripts in a PAO1 strains with an *rsaL* deletion or overproducing RsaL (34, 47) and that the reciprocal control of the *las* and *rhl* QS systems appears to be more complex than predicted in previous studies (56–59). Overall, data showing low heterogeneity in the activation state of the *rhl* system, which is not controlled by an incoherent feed-forward loop, reinforce the importance of the RsaL-dependent negative regulation in determining cell-to-cell variation in the *las* QS activation state. Since RsaL homologs are present in the QS systems of several bacterial species (60), it will be interesting to employ single-cell level analyses to investigate the impact of RsaL on QS activation in bacteria other than *P. aeruginosa* and to clarify if heterogeneity is a common property of QS systems characterized by non-RsaL negative regulators, such as TraM, RsaM, AbaM, and PqsE (61–65).

At present, the significance in *P. aeruginosa* physiology and ecology of maintaining non-quorate cells at high cell density remains unclear. It is known that coexistence of different phenotypic variants in a monoclonal bacterial population could favor division of labor between individuals (66, 67). In other cases, phenotypic heterogeneity could represent a bet-hedging strategy, as part of the population could exhibit a different “pre-adapted” phenotype that will ensure the survival or a better adaptation of part of the bacterial population in case of sudden environmental changes (67, 68). In general, *P. aeruginosa* is an extremely versatile microorganism that integrates a number of environmental signals through multiple regulatory networks for optimally adapting to different niches (69, 70). Among the diverse controlled phenotypes, QS can mediate *P. aeruginosa* transition to different lifestyles (as an example, from the planktonic to the biofilm state, and vice versa), and such transitions could lead individual cells to face consistent and rapid changes in the surrounding environment. It should also be considered that the synthesis of QS signal molecules and QS-regulated exoproducts imposes a metabolic burden on *P. aeruginosa* (71, 72) and that QS activation provides a fitness benefit to this bacterium mainly at high cell density (73). In this regard, maintaining a fraction of cells in a non-quorate state at high cell density could be beneficial if sudden changes in environmental conditions occur, including dilution of the bacterial population. Indeed, non-quorate cells could be better adapted than quorate cells at low cell density, as they could avoid a lag in the shut-down of 3OC_12_-HSL production if suddenly diluted, hence limiting short circuiting of the *las* QS system, i.e., intracellular self-activation of LasR (41). It should be also considered that, even if non-quorate cells are still able to respond to QS signal molecules, high levels of RsaL in non-quorate cells could limit their metabolic burden, as RsaL directly represses the expression of several LasR-dependent genes other than *lasI* (34, 36, 44, 47).

Notably, despite frequent isolation of *P. aeruginosa* clinical and environmental isolates with *lasR* inactivating mutations and/or rewiring of the QS hierarchy (58, 74–76), the *rsaL* gene sequence is conserved among *P. aeruginosa* strains (77), suggesting that the negative regulatory loop generated by RsaL may be important for *P. aeruginosa* fitness in both clinical and natural settings.

## MATERIALS AND METHODS

### Bacterial strains and culture media

Bacterial strains used in this study are listed in Table S1. Bacteria were routinely grown at 37°C in LB with shaking (200 rpm) or on LB plates supplemented with 1.5% (wt/vol) agar (78). When required, the following molecules were added to the medium: gentamicin (Gm, 20 µg/mL for *E. coli*), kanamycin (Km, 30 µg/mL for *E. coli*), tetracycline (Tc, 20 µg/mL for *E. coli* and 200 µg/mL for *P. aeruginosa*), synthetic 3OC_12_-HSL (10 µM for *P. aeruginosa*), and MOPS (50 mM for *P. aeruginosa*). Synthetic 3OC_12_-HSL stock solution was prepared in ethyl acetate acidified with 0.1% (vol/vol) acetic acid at 10 mM concentration.

### DNA manipulation

Restriction enzyme digestions, agarose gel electrophoresis, and ligations were performed using standard methods (78). Transformation of *E. coli* and *P. aeruginosa* was carried out by electroporation (79, 80). Plasmid DNA was purified from bacterial cultures using the Wizard Plus SC Minipreps DNA Purification System (Promega), according to the manufacturer’s instructions. PCR amplifications were performed using GoTaq Polymerase (Promega). FastDigest restriction enzymes were purchased from Thermo Fisher Scientific. Ligation of DNA fragments was performed with the T4 DNA Ligase (Promega). Integrative plasmids were transferred from *E. coli* S17.1λ*pir* donors to *P. aeruginosa* recipient strains by conjugation (78).

### Plasmid construction

Plasmids and oligonucleotides used in this work are listed in Table S2 and Table S3, respectively. The mini-CTX1-derivative plasmids mini-CTX-*gfp* and mini-CTX-*mCherry* were generated as follows: the *gfp* and the *mCherry* genes were PCR amplified with primer pairs *gfp*_FW-*gfp_*RV and *mCherry*_FW-*mCherry*_RV, respectively, using the pRGC vector as template (81) and cloned into the mini-CTX1 plasmid (82) by SmaI-EcoRI or SalI-HindIII, respectively.

The *lasI* promoter region was amplified from *P. aeruginosa* PAO1 genome with primer pairs P*lasI::gfp_*FW-P*lasI::gfp*_RV or P*lasI::mCherry*_FW-P*lasI::mCherry*_RV. The resulting amplicons were cloned by BamHI-SmaI in mini-CTX-*gfp* or by EcoRI-SalI in mini-CTX-*mCherry* to generate mP*lasI::gfp* and mP*lasI::mCherry*, respectively.

The *rhlI* promoter region was amplified from *P. aeruginosa* PAO1 genome with primer pairs P*rhlI::gfp_*FW-P*rhlI::gfp*_RV and cloned by BamHI-SmaI in mini-CTX-*gfp* to generate mP*rhlI::gfp*.

For the generation of the double reporter plasmid mP*lasI::gfp*-P*rsaL::mCherry*, the P*rsaL* promoter region was amplified with primers P*rsaL::mCherry*_FW-P*rsaL::mCherry*_RV and cloned upstream of *mCherry* in the pRGC vector *via* XhoI-ApaI. The DNA fragment corresponding to the P*rsaL::mCherry* fusion was then sub-cloned into the mP*lasI::gfp* plasmid by XhoI-KpnI to obtain mP*lasI::gfp*-P*rsaL::mCherry*.

For the generation of the mP*lasI::sacB* and mPlasI*::*gfp* plasmids, synthetic DNA sequences corresponding to the P*lasI::sacB* fusion and to the promoter variant PlasI* were purchased from Genewiz. Sequences were independently included in the pUC-GW vector, flanked by suitable restriction sites (BamHI-XhoI for P*lasI::sacB*, BamHI-SmaI for PlasI*) for sub-cloning in mini-CTX1 (for the P*lasI::sacB*) or in mini-CTX-*gfp* (for the PlasI* promoter).

All the constructs have been verified by restriction analysis and sequencing.

### Growth conditions and confocal microscopy imaging

A pre-culture procedure was adopted to avoid carryover of GFP or mCherry accumulated in the reporter strains during overnight growth. Briefly, optical density (OD_600_) from overnight cultures was measured, and bacterial suspensions were diluted to allow initial inoculation of 5mL LB-MOPS at OD_600_ of 0.0001. Cultures were incubated at 37°C with shaking (200 rpm) for 5 h, until reaching OD_600_ ≈0.1, washed in LB-MOPS, and diluted into 10 mL of fresh LB-MOPS in 100 mL flasks at an OD_600_ of 0.01. The resulting cultures were incubated at 37°C with shaking. In this way, the PAO1 and ∆*rsaL* reporter strains were in the QS “OFF” state at the beginning of the experiment, showing promoter activities indistinguishable from those observed in the ΔQS reporter strain, in which QS systems are inactive.

For the analyses of phenotypic heterogeneity, aliquots of each culture were taken at the indicated time points for OD_600_ measurement and confocal microscopy imaging. Five-microliter aliquots of each bacterial culture were spotted on a microscope glass slide covered with 0.5% (wt/vol) agarose. Microscopy was performed with a laser scanning confocal microscope Nikon A1R+, using 40× or 100× oil immersion objectives. For each strain, at each analyzed point of the growth curve, images of at least 10 random fields (>750 cells for each experiment) were acquired both in brightfield and fluorescence channels using the following parameters: λ_ex_ 488 nm for GFP and λ_ex_ 561 nm for mCherry. Fluorescence signals emitted by the ΔQS mutant carrying the P*lasI::gfp*, P*lasI::mCherry*, PlasI*::*gfp*, P*rsaL::mCherry*, or P*rhlI::gfp* fusions were used to establish a threshold value below which the promoters can be considered inactive (baseline). The NIS-elements software was used to acquire and pre-process the images (e.g., generation of images with single or overlapping acquisition channels, merge).

Since production of the fluorescent siderophore pyoverdine in *P. aeruginosa* is modulated by the *las* QS system (83, 84), a control experiment has been performed by comparing fluorescence emission in the GFP channel from single cells of PAO1 and its isogenic ∆*pvdA* mutant, impaired in pyoverdine synthesis (85, 86). No fluorescence emission was detectable in the tested strains with our experimental settings (Fig. S6), thus excluding the possible contribution of pyoverdine to the fluorescent signals recorded in the *P. aeruginosa* strains carrying the P*lasI::gfp* transcriptional fusion.

### Quantification of the quorate cell fraction *via* the P*lasI*::*sacB* reporter system

Cultures of wild-type *P. aeruginosa* PAO1 and its isogenic Δ*rsaL* and ΔQS mutants carrying the P*lasI::sacB* transcriptional fusion were incubated at 37°C with shaking (200 rpm) in 10 mL of LB-MOPS in 100 mL flasks until reaching stationary phase (i.e., 6 h of growth). To avoid SacB carryover, the pre-culture procedure described for fluorescent reporter strains was used. At this point, cultures were normalized at OD_600_ of 1, and 1:100 dilutions were incubated for 1 h in 2.5 mL of sterile saline (0.9% [wt/vol] NaCl) supplemented or not with 10% (wt/vol) sucrose. Subsequently, 1:10 serial dilutions were plated on LB agar plates. Plates were incubated at 37°C for 16 h. CFU counts from PAO1, Δ*rsaL*, and ΔQS cultures treated with sucrose were divided to CFU counts from the corresponding untreated cultures to obtain a CFU ratio that represents the fraction of cells which survived sucrose treatment (i.e., the fraction of non-quorate cells). The fraction of quorate cells was calculated as 1 − fraction of non-quorate cells.

### Single-cell image analysis

Quantification of fluorescence in single cells was performed as previously described (19), with minor modifications. ImageJ software was used to analyze the confocal microscopy images and to quantify fluorescence signals from single cells. First, the “analyze particles” command was used to identify single cells on the brightfield images. This allowed generation of a region of interest (ROI) for each cell. Obtained ROIs were overlaid onto the corresponding fluorescent images, so that each single cell identified by an ROI was associated with its own fluorescence intensity. The ImageJ’s “measure” command was used to find pixel intensity within each ROI. Pixel intensity divided by ROI area (herein defined as fluorescence intensity) was used as a proxy for QS activation level. Fluorescence intensity values are reported as percentages relative to the maximum level of fluorescence intensity measured from each reporter strain, considered as 100%.

To quantify the level of heterogeneity for each reporter strain at each time point, the CV (14, 16) in gene expression was determined. CVs have been calculated by dividing the standard deviation obtained from fluorescence intensity values of 750 cells by the mean of the same fluorescence intensity values (the experiment was performed in triplicate). Therefore, high levels of heterogeneity correspond to high CV values, and vice versa.

### Measurement of fluorescence emission in bulk populations

*P. aeruginosa* biosensor strains were grown as indicated for the pre-culture procedure. After that, cultures were washed twice with sterile saline and diluted to an OD_600_ of 0.01. At the indicated time points during bacterial growth, aliquots of 200 µL were taken and dispensed into 96-well black clear-bottom microtiter plates for fluorescence measurement by using an automated luminometer-spectrometer Spark 10M (Tecan). For GFP and mCherry fluorescence measurement, excitation wavelengths of 466 nm and 565 nm and emission wavelengths of 511 nm and 610 nm have been used, respectively (fixed bandwidth, ±10 nm). Fluorescence is given as relative fluorescent units (RFU) divided by OD_600_. Fluorescence was also recorded in the GFP channel for PAO1 and ∆*pvdA* cultures (Fig. S6) to exclude possible contribution of pyoverdine fluorescence to the P*lasI::gfp* fluorescent signal.

### Statistical analysis

Statistical analysis was performed using the GraphPad Prism 6.01 software (https://www.graphpad.com/). Specifically, the one-way analysis of variance test followed by Tukey-Kramer post-hoc test (multiple comparisons) was used for comparisons between three or more groups, while the unpaired *t*-test (single comparison) was used for comparisons between two groups. In both cases, *P* values lower than 0.05 were considered statistically significant. The results of the statistical analysis were reported as means ± SD and were graphically represented as mean ± SD bars in the corresponding figures. The association between GFP and mCherry fluorescence intensities was evaluated using the open-source Orange software (87). This analysis involved computing the Pearson linear correlation coefficient (ρ) and examining the 2D scatter plot of GFP versus mCherry activity using a k-means clustering algorithm (88), the optimal number of clusters being determined by maximizing the silhouette score, a metric that assesses the quality of clustering results based on the separation of data points within and between clusters (89).

## SUPPLEMENTAL MATERIAL

Supplemental figures and tables mbio.02039-23-s0001.pdf Figures S1 to S6 and Tables S1 to S3.

ASM does not own the copyrights to Supplemental Material that may be linked to, or accessed through, an article. The authors have granted ASM a non-exclusive, world-wide license to publish the Supplemental Material files. Please contact the corresponding author directly for reuse.
