# Supplementary material for: RsaL-driven negative regulation promotes heterogeneity in Pseudomonas aeruginosa quorum sensing
Source: mBio. 2023 Oct 16;14(6):e02039-23. doi: 10.1128/mbio.02039-23 (PMC10746200; doi:10.1128/mbio.02039-23)
Supplement: Supplemental figures and tables — Figures S1 to S6 and Tables S1 to S3. [file mbio.02039-23-s0001.pdf]

RsaL-driven negative regulation promotes heterogeneity in *Pseudomonas aeruginosa* quorum sensing

Marta Mellini[a#], Morgana Letizia[a], Lorenzo Caruso[a], Alessandra Guiducci[a], Carlo Meneghini[a], Stephan Heeb[b], Paul Williams[b], Miguel Cámara[b], Paolo Visca[a,c,d], Francesco Imperi[a,c,d], Livia Leoni[a], Giordano Rampioni[a,d#]

[a] *Department of Science, University Roma Tre, Rome, Italy*

[b] *National Biofilms Innovation Centre, Biodiscovery Institute and School of Life Sciences, University of Nottingham, Nottingham, United Kingdom*

[c] *NBFC, National Biodiversity Future Center, Palermo, Italy*

[d] *IRCCS Fondazione Santa Lucia, Rome, Italy*

Supplemental Information

Figure S1. RsaL influences the proportion of cells activating the P*lasI*::*mCherry* fusion

Figure S2. RsaL influences the distribution of cells activating the P*lasI*::*gfp* fusion

Figure S3. RsaL increases heterogeneity of P*lasI*::*mCherry* activity and influences the distribution of cells activating the P*lasI*::*mCherry* fusion

Figure S4. The *P. aeruginosa rhl* QS system

Figure S5. RsaL does not influence the distribution of cells activating the P*rhlI*::*gfp* fusion

Figure S6. Pyoverdine does not contribute to the fluorescence signals in the tested conditions

Table S1. Bacterial strains used in this study

Table S2. Plasmids used in this study

Table S3. Oligonucleotides used in this study

References

Figure S1

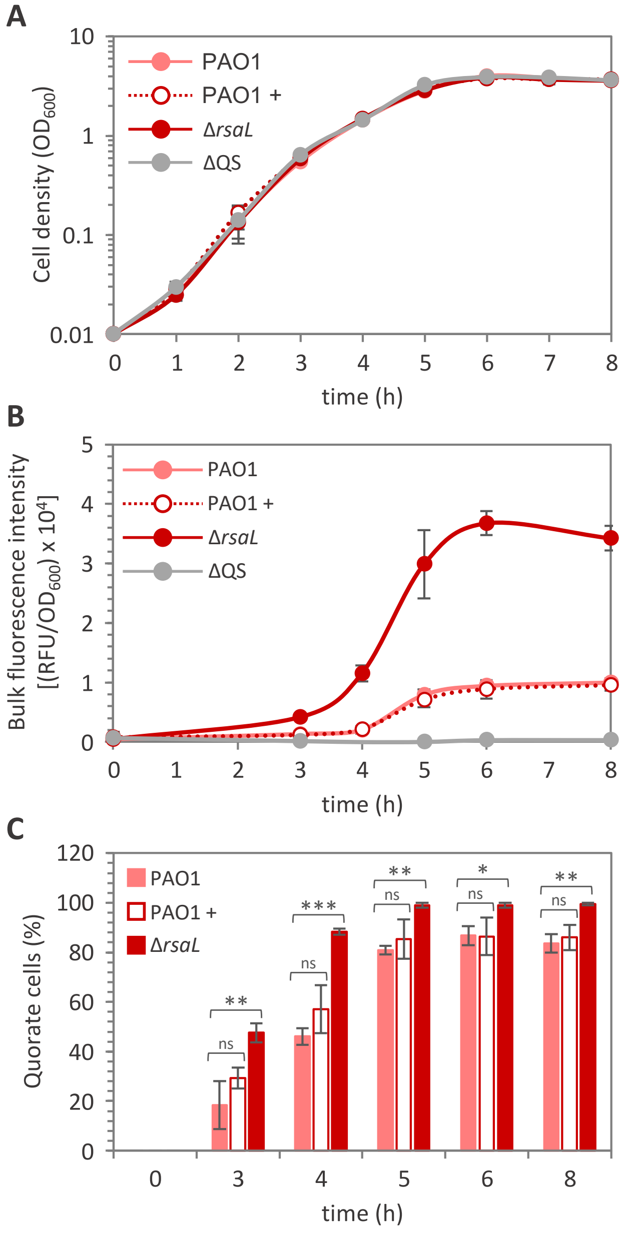

Figure S1. RsaL influences the proportion of cells activating the P*lasI*::*mCherry* fusion. (A) Growth curves, (B) bulk population analyses of P*lasI* activity, and (C) percentage of cells with active P*lasI* (quorate cells) in cultures of PAO1 incubated (+) or not with 10 µM 3OC$_{12}$-HSL, and of the indicated isogenic mutants, all carrying the P*lasI*::*mCherry* fusion integrated into the chromosome. For (A) and (B), means and standard deviations were obtained from three independent experiments. For (C), the count of quorate cells for each sample at each time point was conducted on 2,250 cells from three biological replicates (750 cells each). *, $P < 0.05$; **, $P < 0.01$; ***, $P < 0.001$; ns, not statistically significant.

Figure S2

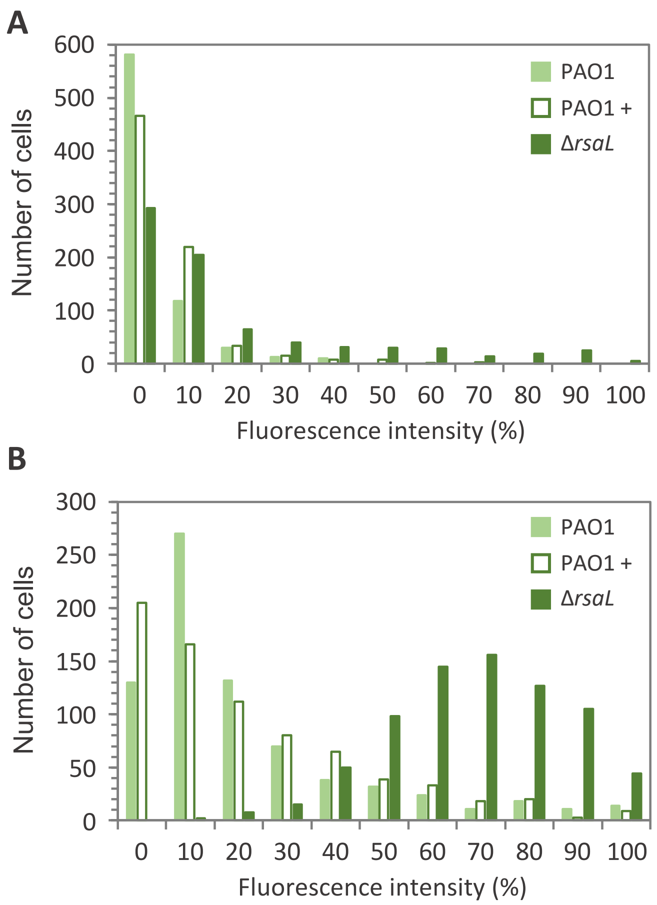

Figure S2. RsaL influences the distribution of cells activating the P*lasI::gfp* fusion. Histograms reporting fluorescence distributions from single cells of PAO1 incubated (+) or not with 10 µM $3OC_{12}$-HSL and its isogenic mutant Δ*rsaL*, both carrying the P*lasI::gfp* fusion integrated into the chromosome. Data refer to cultures grown to (A) low cell density (*i.e.*, 3 h of growth), and (B) high cell density (*i.e.*, 8 h of growth). For each biological replicate and at each time point, fluorescence quantification has been conducted on 750 cells per strain/condition. A representative dataset from one out of three biological replicates is shown (same dataset as for Fig. 4A).

Figure S3

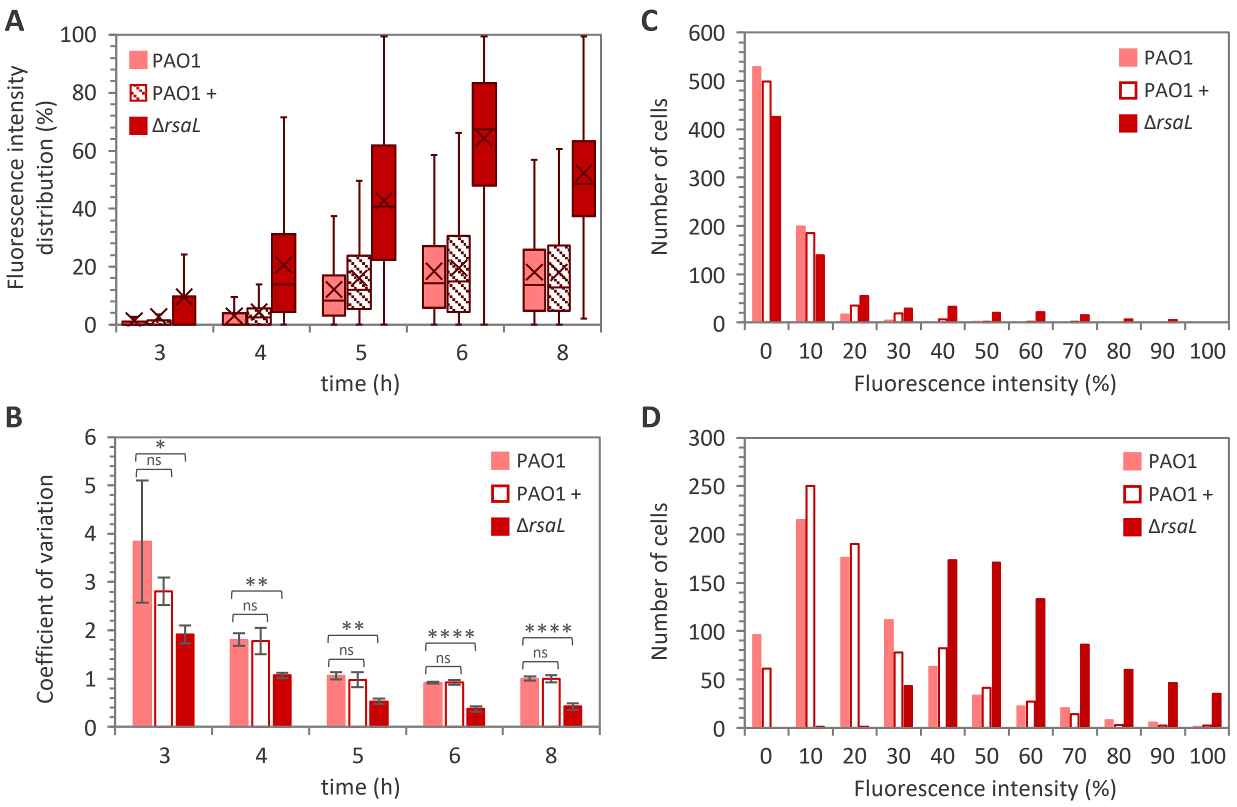

Figure S3. RsaL increases heterogeneity of P*lasI*::*mCherry* activity and influences the distribution of cells activating the P*lasI*::*mCherry* fusion. (A) Box plot graph showing fluorescence distribution and (B) coefficient of variation from single cells of PAO1 incubated (+) or not with 10 µM $3OC_{12}$-HSL and its isogenic Δ*rsaL* mutant, both carrying P*lasI*::*mCherry* fusion integrated into the chromosome. (C-D) Histograms reporting fluorescence distributions from single cells of PAO1 incubated (+) or not with 10 µM $3OC_{12}$-HSL and its isogenic mutant Δ*rsaL*, both carrying the P*lasI::mCherry* fusion integrated into the chromosome. Data refer to cultures grown to (C) low cell density (*i.e.*, 3 h of growth), and (D) high cell density (*i.e.*, 8 h of growth). For (A), (C) and (D), fluorescence intensity is given as % relative to the Δ*rsaL* P*lasI*::*mCherry* cell showing the highest fluorescence intensity after 8 h of growth, considered as 100%. In (A), mean P*lasI* activity derived from fluorescence intensity values measured in single cells is indicated with an X in the box plots. The horizontal lines in the box plots represent the median values. For (A), (C) and (D), for each biological replicate and at each time point, fluorescence quantification has been conducted on 750 cells per strain/condition. A representative dataset from one out of three biological replicates is shown. For (B) means and standard deviations were obtained from three biological replicates (750 cells each). *, $P < 0.05$; **, $P < 0.01$; ****, $P < 0.0001$; ns, not statistically significant.

Figure S4

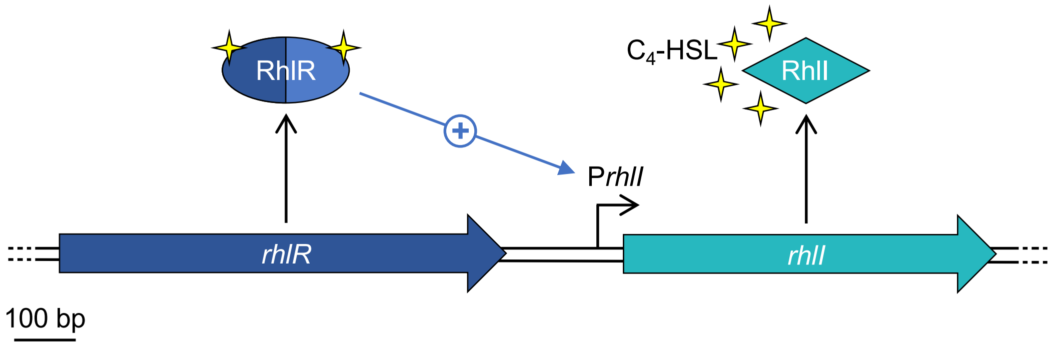

Figure S4. The *P. aeruginosa rhl* QS system. Schematic representation of the DNA region encompassing the *rhlR* and *rhlI* genes in *P. aeruginosa* [S1]. The solid blued arrow indicates activation (+).

.

Figure S5

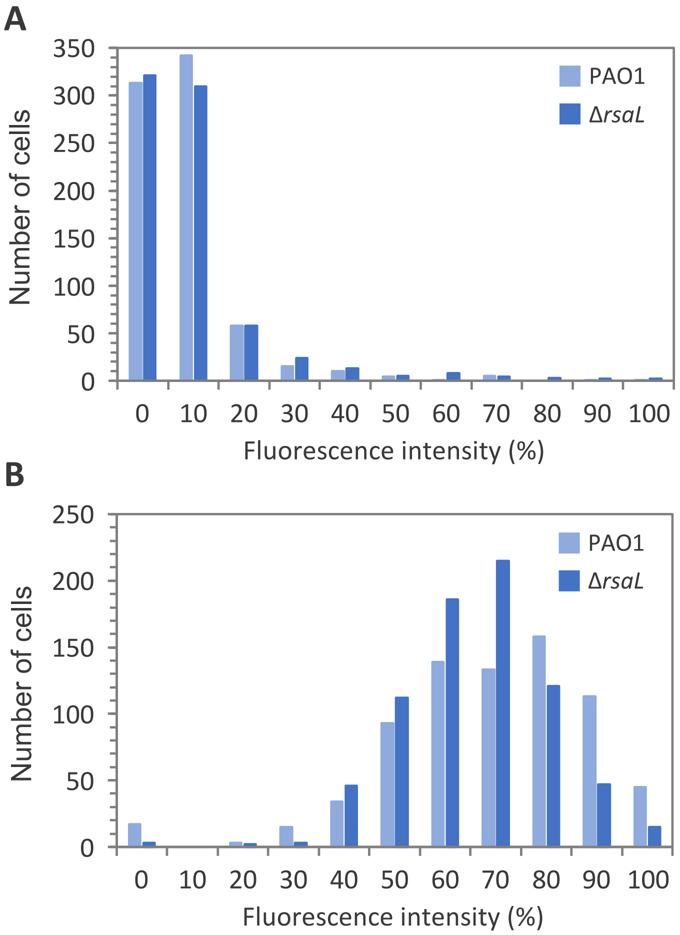

Figure S5. RsaL does not influence the distribution of cells activating the P*rhlI*::*gfp* fusion. Histograms reporting fluorescence distributions from single cells of PAO1 incubated (+) or not with 10 µM 3OC$_{12}$-HSL and its isogenic mutant Δ*rsaL*, both carrying the P*rhlI::gfp* fusion integrated into the chromosome. Data refer to cultures grown to (A) low cell density (*i.e.*, 3 h of growth), and (B) high cell density (*i.e.*, 8 h of growth). For each biological replicate and at each time point, fluorescence quantification has been conducted on 750 cells per strain/condition. A representative dataset from one out of three biological replicates is shown (same dataset as for Fig. 7C).

Figure S6

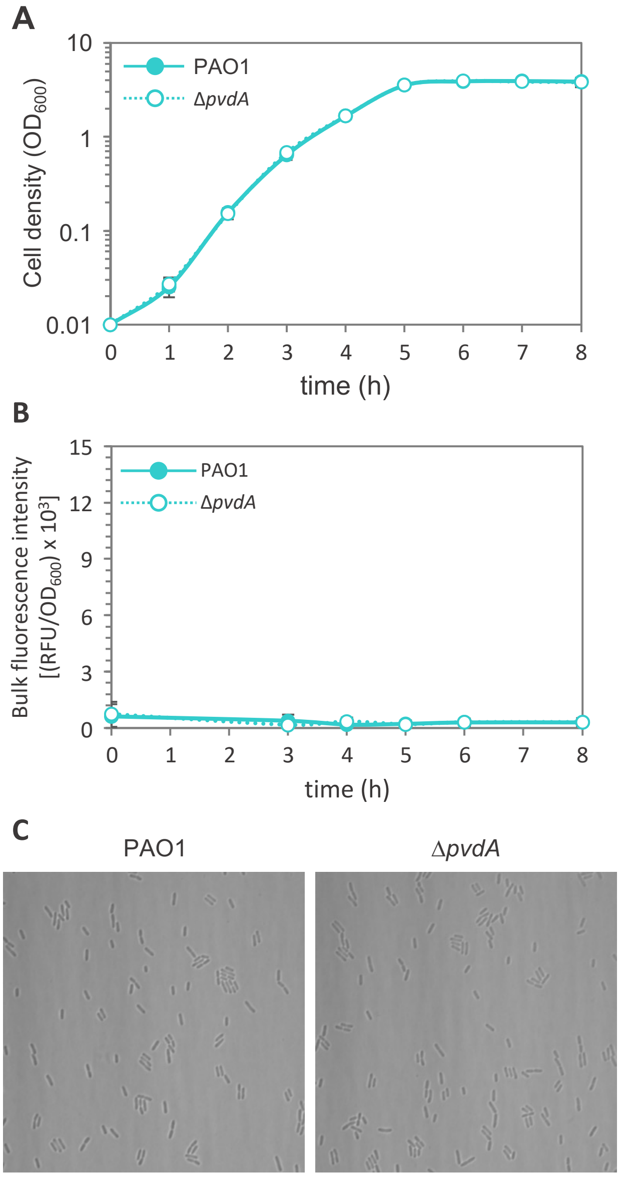

Figure S6. Pyoverdine does not contribute to the fluorescence signals in the tested conditions. (A) Growth curves, (B) bulk population and (C) single cell level analyses of fluorescence in the GFP channel in PAO1 and Δ*pvdA* strains grown in LB-MOPS. The same experimental settings used to obtain the data reported in Fig. 2 were employed in this analysis. For this reason, in (B) the same scale has been used for the *y* axis as in Fig. 2B. For (A) and (B), means and standard deviations were obtained from three independent experiments. For (C), a representative image of stationary phase PAO1 and Δ*pvdA* cells is shown.

Table S1. Bacterial strains used in this study

Strains	Relevant characteristics	Reference/Source
E. coli		
S17.1λ*pir*	Conjugative strain for suicide plasmids.	[S2]
P. aeruginosa		
PAO1	ATCC 15692 type strain	ATCC
Δ*rsaL*	PAO1 derivative strain carrying in frame deletion of the *rsaL* gene	[S3]
ΔQS	PAO1 derivative strain carrying in frame deletions of the *lasR-rsaL-lasI*, *rhlR-rhlI, pqsABCDE-phnAB-pqsR* gene *loci*, and of the *pqsH* and *pqsL* genes.	[S4]
Δ*pvdA*	PAO1 derivative strain carrying in frame deletions of the *pvdA* gene.	[S5]

Table S2. Plasmids used in this study

Plasmids	Relevant characteristics and plasmids construction	Reference/Source
mini-CTX1	Suicide vector for site-specific integration in *P. aeruginosa* chromosome; Tc^R.	[S6]
pRGC	*E. coli-Pseudomonas* sp. promoter probe shuttle vector for transcriptional fusion carrying *mCherry, gfp* and *cfp* promoterless genes; source of *gfp* and *mCherry* genes; Gm^R.	[S7]
mini-CTX-*gfp*	mini-CTX1 derivative promoter probe plasmid for transcriptional fusion carrying the promoterless *gfp* gene; Tc^R.	This study
mini-CTX-*mCherry*	mini-CTX1 derivative promoter probe plasmid for translational fusion carrying the promoterless *mCherry* gene; Tc^R.	This study
mP*lasI*::*gfp*	mini-CTX-*gfp* derivative carrying the P*lasI* promoter upstream *gfp*; Tc^R.	This study
mP*lasI*::*mCherry*	mini-CTX-*mCherry* derivative carrying the P*lasI* promoter upstream *mCherry*; Tc^R.	This study
pUC-GW-P*lasI*::*sacB*	Plasmid containing the P*lasI*::*sacB* transcriptional fusion; Km^R	Genewiz
mP*lasI*::*sacB*	mini-CTX-1 derivative carrying the P*lasI* promoter upstream the *sacB* gene; Tc^R.	This study
pUC-GW-P*lasI**	Plasmid containing the P*lasI** DNA fragment; Km^R.	Genewiz
mP*lasI**::*gfp*	mini-CTX-*gfp* derivative carrying the P*lasI** promoter upstream *gfp*; Tc^R.	This study
pRGC P*rsaL*::*mCherry*	pRGC derivative carrying the P*rsaL* promoter upstream *mCherry*; Gm^R.	This study
mP*rsaL*::*mCherry*-P*lasI*::*gfp*	mini-CTX-*mCherry-gfp* derivative carrying the P*lasI* promoter upstream *gfp* and the P*rsaL* promoter upstream *mCherry*; Tc^R.	This study
mP*rhlI*::*gfp*	mini-CTX-*gfp* derivative carrying the P*rhlI* promoter upstream gfp; Tc^R.	This study

Table S3. Oligonucleotides used in this study

Name	Sequence (5'-3')[a]	Restriction site
*gfp*_FW	TCCCCCGGGTAAGTAAGTAATTAAAGAGGAGAAATTAAGCATG	SmaI
*gfp*_RV	CCGGAATTCCTCCCTGAAAATCTCGCCAAG	EcoRI
*mCherry*_FW	TATAAGCTTTGGTGAGCAAGGGCGAGG	SalI
*mCherry*_RV	TATGTCGACTCACTTGTACAGCTCGTCCATG	HindIII
P*lasI*::*gfp*_FW	CGCGGATTCGGCTGTGTTCTCTCGTGT	BamHI
P*lasI*::*gfp*_RV	TCCCCCGGGTACGATCATCTTCACTTCCT	SmaI
P*lasI*::*mCherry*_FW	TATGAATTCGGCTGTGTTCTCTCGTGTG	EcoRI
P*lasI*::*mCherry*_RV	TATAAGCTTGTACGATCATCTTCACTTCC	SalI
P*rhlI*::*gfp*_FW	CGCGGATCCCCGATGCTGATGTCCAACC	BamHI
P*rhlI*::*gfp*_RV	TCCCCCGGGACCAAGTCCCCGTGTCG	SmaI
P*rsaL*::*mCherry*_FW	CCGCTCGAGCCAATTTGTACGATCATCTTCA	XhoI
P*rsaL*::*mCherry*_RV	ATAGGGCCCGGCTGTGTTCTCTCGTGT	ApaI

[a] Restriction sites are underlined in the primer sequences.

References

S1. Winsor GL, Lam DK, Fleming L, Lo R, Whiteside MD, Yu NY, Hancock RE, Brinkman FS. 2011. *Pseudomonas* Genome Database: improved comparative analysis and population genomics capability for *Pseudomonas* genomes. Nucleic Acids Res 39:D596-600.

S2. Simon R, Priefer U, Puhler A. 1983. A broad host range mobilization system for *in vivo* genetic-engineering: transposon mutagenesis in Gram-negative bacteria. Biotechnology 1:784-791.

S3. Bondí R, Longo F, Messina M, D'Angelo F, Visca P, Leoni L, Rampioni G. 2017. The multi-output incoherent feedforward loop constituted by the transcriptional regulators LasR and RsaL confers robustness to a subset of quorum sensing genes in *Pseudomonas aeruginosa.* Mol Biosyst 13:1080-1089.

S4. Letizia M, Mellini M, Fortuna F, Visca P, Imperi F, Leoni L, Rampioni G. 2022. PqsE Expands and Differentially Modulates the RhlR Quorum Sensing Regulon in *Pseudomonas aeruginosa*. Microbiol Spectr 10:e0096122.

S5. Imperi F, Putignani L, Tiburzi F, Ambrosi C, Cipollone R, Ascenzi P, Visca P. 2008. Membrane-association determinants of the omega-amino acid monooxygenase PvdA, a pyoverdine biosynthetic enzyme from *Pseudomonas aeruginosa*. Microbiology (Reading) 154:2804-2813.

S6. Hoang TT, Kutchma AJ, Becher A, Schweizer HP. 2000. Integration-proficient plasmids for *Pseudomonas aeruginosa*: site-specific integration and use for engineering of reporter and expression strains. Plasmid 43:59-72.

S7. Mellini M, Lucidi M, Imperi F, Visca P, Leoni L, Rampioni G. 2021. Generation of Genetic Tools for Gauging Multiple-Gene Expression at the Single-Cell Level. Appl Environ Microbiol 87:e02956-20.
